# Using intracellular plasmonics to characterize nanomorphology in human cells

**DOI:** 10.1038/s41378-020-00219-w

**Published:** 2020-12-14

**Authors:** Ahmad Sohrabi Kashani, Alisa Piekny, Muthukumaran Packirisamy

**Affiliations:** 1 Optical Bio-Microsystem Lab, Micro-Nano-Bio-Integration Center, Department of Mechanical, Industrial and Aerospace Engineering, Concordia University, 1455 De Maisonneuve Blvd. W., Montreal, QC H3G 1M8 Canada; 2 Department of Biology, Concordia University, 7141 Sherbrooke Street W., Montreal, QC H4B 1R6 Canada

**Keywords:** Nanoparticles, Engineering

## Abstract

Determining the characteristics and localization of nanoparticles inside cells is crucial for nanomedicine design for cancer therapy. Hyperspectral imaging is a fast, straightforward, reliable, and accurate method to study the interactions of nanoparticles and intracellular components. With a hyperspectral image, we could collect spectral information consisting of thousands of pixels in a short time. Using hyperspectral images, in this work, we developed a label-free technique to detect nanoparticles in different regions of the cell. This technique is based on plasmonic shifts taking place during the interaction of nanoparticles with the surrounding medium. The unique optical properties of gold nanoparticles, localized surface plasmon resonance bands, are influenced by their microenvironment. The LSPR properties of nanoparticles, hence, could provide information on regions in which nanoparticles are distributed. To examine the potential of this technique for intracellular detection, we used three different types of gold nanoparticles: nanospheres, nanostars and Swarna Bhasma (SB), an Indian Ayurvedic/Sidha medicine, in A549 (human non-small cell lung cancer) and HepG2 (human hepatocellular carcinoma) cells. All three types of particles exhibited broader and longer bands once they were inside cells; however, their plasmonic shifts could change depending on the size and morphology of particles. This technique, along with dark-field images, revealed the uniform distribution of nanospheres in cells and could provide more accurate information on their intracellular microenvironment compared to the other particles. The region-dependent optical responses of nanoparticles in cells highlight the potential application of this technique for subcellular diagnosis when particles with proper size and morphology are chosen to reflect the microenvironment effects properly.

## A finer way to look inside cells

Researchers in Canada have demonstrated a technique to gather high-resolution information about nanoparticles distributed within cells. The team, led by Muthukumaran Packirisamy of Concordia University, used hyperspectral imaging combined with dark-field imaging to detect nanoparticles injected into human cancer cells. The technique is quick and does not require that the nanoparticles be labeled. Interactions between the nanoparticles and their microenvironment affect the optical responses of the nanoparticles, making it possible to use them to detect differences between subcellular regions or compartments. The team tested the approach with three types of gold nanoparticles: nanospheres, nanostars, and an Ayurvedic medicine known as Swarna Bhasma. They found that nanospheres performed better than nanostars or Swarna Bhasma. By selecting particles of the appropriate size and morphology, this imaging technique can be a powerful tool for subcellular diagnosis.

## Introduction

Nanoparticles (NPs) with different physicochemical properties are being developed for a wide range of biomedical applications. Due to their small size and tunability, they are being explored for use in cancer diagnostics and treatment. NPs can be used as carriers to deliver drugs with greater efficiency than conventional methods. For example, with an NP delivery system, the release of drugs can be optimized under controllable conditions. The lack of selectivity and negative effects on healthy tissues/cells are the main drawbacks of classical methods^1–3^. NPs enable an advanced approach to developing therapeutic techniques in which NPs with unique physicochemical properties, nanomedicines, are designed to transport drugs to a specific place in the body or even in the cells. Before exploring their clinical use, it is essential to thoroughly evaluate NPs in cells to understand their toxicity and distribution and how they impact the intracellular environment and vice versa^4,5^. Detection and intracellular localization are critical steps in the development of nanomedicines and are extremely challenging because of the sensitivity and resolution limits of the current techniques. To understand nanomedicine-based treatment efficiency, we need to advance our ability to visualize NP uptake and identify/characterize NPs at the subcellular level^6–8^.

Various microscopic and spectroscopic techniques have been developed for the subcellular detection and visualization of NPs. Scanning electron microscopy (SEM) and transmitting electron microscopy (TEM) have high resolution and permit the visualization of NPs at the subcellular level. For example, Shapero et al.^9^ used TEM to visualize silica particles in A549 cells and found that these NPs are localized in lysosomes and not in the nucleus. Despite the advantages of TEM, this technique is costly and requires complicated sample preparation that can impact cellular structures^10^. Sample preparation for SEM is simple; however, this technique can image only the surface of cells. Light microscopy can be used to visualize both NPs and their surrounding environment; however, only clusters of particles can be visualized^5^. Recently, Chen et al.^11^ developed a superresolution fluorescence imaging technique with a spatial resolution of 10 nm, well below the light diffraction limit. They used this technique to detect silica nanoparticles in HeLa cells, which they visualized in lysosomes and mitochondria. However, this method has limitations because it requires the addition of fluorescent tags onto NPs, which is restricted by their functionalization and requires a system capable of superresolution imaging.

Raman spectroscopy (RS) and infrared absorption (IR) have also been used to detect and localize NPs at the subcellular level^12^. The low spatial resolution of the IR technique has limited the application of this technique for intracellular diagnosis. Raman spectroscopy is a powerful technique and can differentiate chemical structures in cells based on their unique vibrational modes. Once a laser illuminates a sample, the emissions are shifted by the vibrational properties of molecules due to inelastic scattering^10,13,14^. Noble NPs such as gold can enhance the intensity of Raman spectra in the vicinity of molecules, enabling the technique called surface-enhanced Raman spectroscopy (SERS). With SERS effects, the localization/distribution of NPs at the subcellular level can be determined by Raman scanning over the entire cell^15^. The difficulty in interpreting the SERS spectra, and the time-consuming operation for Raman scanning, are two main limitations of Raman techniques^16,17^.

Recently, our group monitored the intracellular location of NPs using plasmon properties^18^. For this technique, hyperspectral images (HSIs) in combination with dark-field imaging (DK) are utilized to characterize different subcellular components and approximate the locations of NPs. Over the past few years, hyperspectral imaging techniques have received considerable attention for single-cell analysis^19–23^. Both spectral and spatial information on NPs can be simultaneously recorded with hyperspectral imaging, providing a novel and label-free platform for the intracellular characterization of NPs. The entire spectrum can be acquired at each point in the HSI image, and in contrast to the SERS method^24,25^, no complex interpretation is required. With the HSI technique, high-resolution spectral information consisting of thousands of pixels can be recorded in less than one hour. With this technique, the localized surface plasmon resonance (LSPR) of gold and silver can be studied at the nanoscale to estimate their location and monitor their microenvironment in cells. The LSPR properties of NPs depend on their physicochemical properties, such as size, morphology, functionalization, and the surrounding medium. Once metallic nanoparticles are in cells, they interact with intracellular components. Hence, the particles are surrounded by biological environments with specific dielectric constants, resulting in core-shell nanohybrids. The optical properties of these nanohybrids are changed compared to those of the core parts (metallic particles)^26–28^. The hyperspectral technique could provide information on these changes and on their intracellular localization. NPs can be characterized in cells much more rapidly using this advanced label-free method instead of SERS, while NP internalization can be studied by measuring unique spectral signatures.

**Fig. 1:**
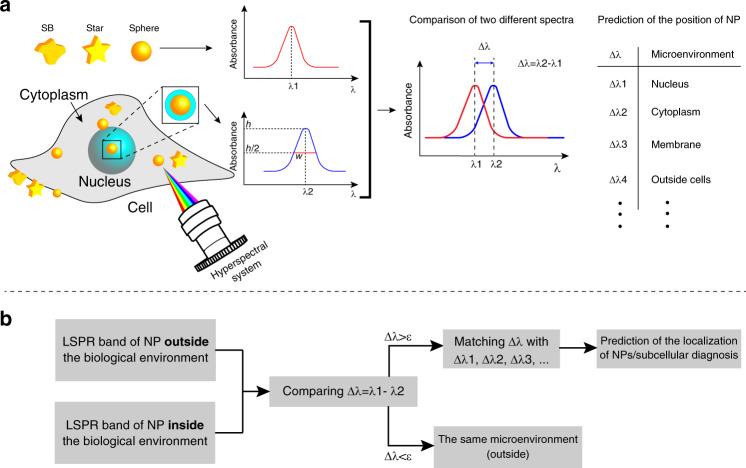
Subcellular diagnosis using hyperspectral images and plasmonic shift. **a** A schematic showing how the detection/localization of NPs (with different morphologies) is determined within cells by the hyperspectral technique when their surrounding microenvironments are changed. **b** The concept of using hyperspectral images for subcellular detection/localization (ε shows very small differences in the LSPR band)

Here, in the current paper, we used hyperspectral imaging to study the interactions of three different morphologies of gold NPs: nanospheres, nanostars, and Swarna Bhasma (SB), an Indian Ayurvedic/Sidha medicine, in A549 (human non-small cell lung cancer) and HepG2 (human hepatocellular carcinoma) cells. SBs are large gold-derived particles prepared as Ayurvedic/Sidha medicine and are variable in different compositions and shapes^29,30^. Three different aspects were studied in this work: (1) nanoparticle characterization in cells, (2) hyperspectral techniques for intracellular sensing, and (3) how nanomorphology could affect intracellular sensing. As described earlier, the LSPR of gold particles is sensitive to the surrounding medium, and we measured changes in plasmonic shifts to estimate the intracellular location and characteristics of particles in cells (Fig. 1). In addition, with this system, we could confirm the presence of NPs in cells by considering their unique spectral signatures. Our findings showed that the distribution of each type of gold particle is different. A greater accumulation of nanospheres than of other types of particles was observed in cells, and the nanospheres exhibited a greater plasmonic shift with higher sensitivity to their neighboring medium. This study revealed that the intracellular plasmon depends on the size and morphology of particles. The proposed subcellular detection method will help enhance our understanding of nano-bio-interaction and develop nanomedicines for therapeutic and diagnostic applications.

## Materials and methods

### Synthesis of gold nanostars and nanospheres

The Turkevich method^31^, the most commonly used technique for preparing spherical particles, was used to synthesize nanospheres by reducing gold chloroauric acid to gold atoms. Briefly, we boiled 80 mL of 40 µg/mL HAuCl_4_.3H_2_O solution and then added 5 mL of 1% sodium citrate. We then heated the solution for 15 min, and the solution changed from pale yellow to purple and then ruby-red. For the preparation of nanostars, a seed-mediated approach was utilized. Ten milliliters of the 0.25 mM gold solution was mixed with 10 µL of 1 M HCl and 100 µL of presynthesized seed solution. Ascorbic acid and silver nitrate were then simultaneously added to the solution and stirred for a few minutes. Then, the solution became blue.

### Swarna bhasma

Swarna Bhasma (SB) particles are Indian gold-based particles that are prepared by the incineration of gold in the presence of herbal extracts. The incineration process is performed many times until their size reaches that of the chemically synthesized particles^29,30^. SB particles were purchased from Jaya Indian Medicine Pharmaceutical Pvt Ltd, Maduravoyal, Chennai, Tamilnadu, India in powder format, and they were suspended in deionized water.

### Hyperspectral imaging system and processing data

**Fig. 2:**
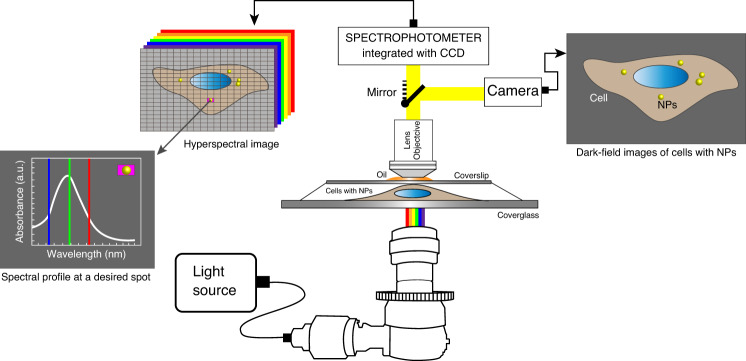
Dark-field microscopy coupled to hyperspectral imaging to study gold nanoparticles in cells. A schematic showing the hyperspectral imaging instrument coupled with a dark-field imaging system to study the interaction of nanomaterials with cells.

A hyperspectral imaging system (CytoViva, Aruban, AL, USA) was used to capture HSI images of fixed NP-treated cancer cells. This system provides a high-resolution dark-field-based optical microscope combined with a photospectrometer for spectral characterization and spectral mapping at the nanoscale. The spectrophotometer is connected to a charged-coupled device (CCD) camera attached to a microscope to take images of the sample (Fig. 2). This method is a label-free technique and does not need any labels. This system permits capturing visible to near-infrared spectra in the range of 400–1000 nm, with a resolution of 2.8 nm within each pixel of the scanned field of view. Each pixel of an HSI image is ~25 nm using the ×100 objective. Gold particles are brighter in HSI images, and they can be characterized by studying their spectral information. The LSPR properties of gold particles in the biological environment can be measured to identify and determine their spatial distributions in cells without damaging the sample^32^.

To capture HSI images, the condenser was adjusted until it touched the cover glass in the presence of the oil, and an oil-immersion objective (×60 or ×100) was then used to focus on the desired area. The exposure time for each spectral area was set to 0.15–0.25 s, and the intensity was adjusted to a value between 1000–5000 units to ensure that spectral signals do not exceed the maximum limit. The desired area was scanned at 700 × 700 pixel resolution. We then used ENVI 4.8 (Exelis Visual Information Solutions, Boulder, CO, USA) software for processing the collected spectral data. We chose the desired regions of interest (ROIs), and the mean spectral data were collected for approximately 70–100 pixels. A spectral library (SPL) was created from the ROI, and their spectra were filtered against the control cells (without particles) to obtain the spectral data on gold NPs (F-SPL). Furthermore, the filtered spectral data were calibrated with respect to the source light spectrum. Finally, the spectral intensities were normalized to between 0 and 1 to compare LSPR bands.

Spectral mapping was performed using the spectral angle mapper (SAM) algorithm to scan the HSI images and find pixels whose spectral profile is matched with the F-SPL with a threshold value of 0.1. In this algorithm, the intensity of the spectra does not affect the SAM analysis. The possibility of ignoring the intensity of the signal is an advantage of the SAM, as any variation in spectral intensity does not affect the results. A smaller difference shows a better match with the reference spectrum.

### Particle detection in different regions

We used microscopic techniques to compare the plasmonic responses of particles in three different regions: inside the nucleus, at the periphery of the nucleus, and at the periphery of cells. We identified NPs that are very clearly inside the cells. We focused on the target particles/aggregates to obtain a high-resolution view of the particles, and we then changed the level of the focus to ensure that they are inside and not on the membrane or outside of the cells. Next, we used the ROI tool to find their average LSPR responses. We performed the same process for particles (with almost the same size) that were clearly inside the nucleus and on or outside the perimeter of cells to measure the plasmonic shifts. Note that once particles are aggregated, the dipole-dipole interactions between particles might affect their LSPR response^33^. To minimize the effects of dipole-dipole interactions on our comparison, we tried to compare aggregates with the same size in and out of cells. We applied the same strategy to quantify the uptake of particles in different regions of cells. Considering the size range of each type of particle (measured by DLS), we used ImageJ to count the average number of three different types of NPs in two different cell lines^12^. Student’s *t*-test was used to compare statistical data, and differences were considered significant at *p* < 0.05.

### Cell culture

A549 (human non-small cell lung carcinoma) and HepG2 (human hepatocellular carcinoma) cells were used to characterize gold NPs in cells. A549 cells and HepG2 cells were cultured in F-12 medium and Dulbecco’s modified Eagle’s medium (DMEM; Wisent), respectively. Medium was supplemented with 10 (v/v) fetal bovine serum (FBS; Thermo Fisher Scientific), 2 mM glutamine, 100 µg/mL streptomycin, and 100 units/mL penicillin (Wisent). Cells were kept in an incubator at 37 °C with 5% CO_2_ and passaged at 70% confluency.

### Cell fixation

Cells were plated on acid-washed glass coverslips in 6-well dishes to 40% confluency, and then particles were added for 24-48 h. Cells were fixed in fresh, ice-cold 10% w/v trichloracetic acid (TCA) and washed with TBST as previously described^30^ before placing the coverslip on a slide and sealing.

### Size and zeta potential measurements of particles

We used a Zetasizer Nano ZS90 (Malvern Instruments Ltd., Worcestershire, UK) along with dynamic light scattering (DLS) analysis to measure the average size of all gold particles. The system is equipped with a red laser (633 nm) with a power of 4 mW and a detection angle of 90°. The hydrodynamic size of particles was measured in deionized water based on Brownian motion. Due to the different sizes of particles, light is scattered with different intensities. We added 1 mL of each suspension to a disposable (scratch-free) square cuvette. For each gold sample, we made ten DLS measurements and reported the average sizes in this study. Furthermore, we used a Zeta Plus electrophoresis instrument (Brookhaven Instrument Corp.) to determine the zeta potential of gold particles. The particles were introduced into a chamber containing two electrodes. An electric field was then applied to the electrodes. The particles travel with different velocities toward oppositely charged electrodes, depending on their surface charge. The migration of particles is illuminated with a laser beam, and the velocity is estimated based on the frequency shift in the scattered light. The velocity was measured and correlated to the zeta potential by the Henry equation^34^. For our measurements, we set the temperature and pH at 25 °C and 7.4, respectively. For each sample, we analyzed the sample ten times, and the average was reported as the zeta potential of each type of particle.

## Results and discussion

### Characterization of gold particles

**Fig. 3:**
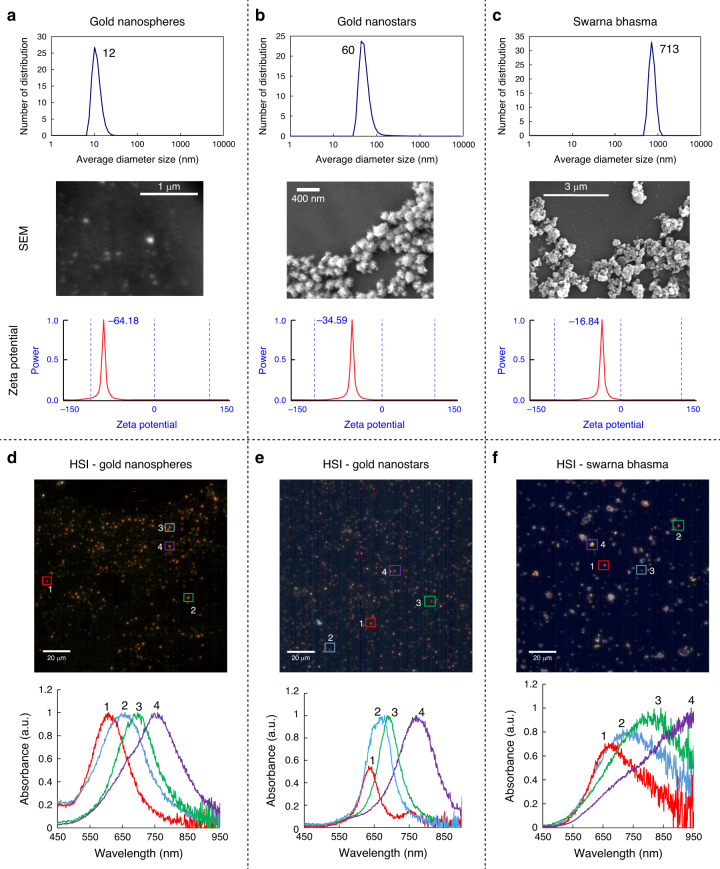
Gold particles characterization using different techniques. **a** Average size of nanospheres measured by DLS, SEM images of nanospheres and their zeta potential. **b** The average size of nanostars (by DLS), SEM of nanostar aggregates, and their zeta potential. **c** Size distribution of SB particles suspended in deionized water (by DLS), SEM of SBs, and their zeta potential, **d** A hyperspectral image (top) of nanospheres and corresponding LSPR bands (bottom). **e** A hyperspectral image (top) of nanostars and corresponding LSPR bands (bottom). **f** A hyperspectral image (top) of SBs and corresponding LSPR bands (bottom)-for HSI image; scale bar is 20 µm

The physicochemical properties of three different types of gold particles were characterized by DLS, SEM, and potential zeta analyzers before using them in cells. DLS showed that the hydrodynamic diameter of the nanospheres is smaller than that of other nanoparticles, with an average size of 12 nm (Fig. 3a). Nanostars, as shown in Fig. 3b, tended to aggregate when dried on glass coverslips, and the size of the individual nanostars varied between 50 and 100 nm with an average size of 60 nm. SB particles were significantly larger than nanostars and nanospheres. The crystal size of SBs was approximately 70 nm^30^, while the average size of the aggregates was up to 700 nm (Fig. 3c). With the use of electrophoretic light scattering, we measured the zeta potential of particles based on the Poisson-Boltzmann equation^35^. All particles displayed a negative potential; however, their levels were different. Synthesized particles showed negative zeta potentials due to the presence of citrate on their surface membrane. During the reduction process, both nanostars and nanospheres are decorated with citrates, which are the main source of the negative charge. Nanospheres showed a greater negative potential than nanostars, suggesting that they are more stable. Zeta potential measurements showed that the surface of SBs was coated with negative ions, but their zeta potential was less negative than that of the other particles. The elemental composition of SBs was reported in our previous studies^29,30^. Gold is the main element in SBs; however, they also contain other elements such as Mg, Ca, Fe, and Si.

### Hyperspectral imaging for particle characterization

The particles were further characterized by hyperspectral imaging. Particles were dried on glass coverslips and sealed, and their LSPR spectra were measured. Depending on their size, the particles have different colors. The LSPR properties for the three different types of gold particles in this study are shown in Fig. 3d–f. SBs and nanostars are larger, with longer-wavelength LSPR bands. Nanospheres are smaller and more consistent in size, with LSPR bands of 560 nm for individual particles/small aggregates and between 650–750 nm for larger aggregates (Fig. 3d). Nanostars had two different peaks, one strong peak reflecting their spherical core and another weak peak corresponding to the branches (Fig. 3e). The band at 750 nm (particle #1) belongs to branches, which become stronger when they get longer. The weaker signals confirm that branches are short, as shown in the SEM images. SB particles do not have any specific shape, and their LSPR bands are broader than those of the other particles. Smaller SBs have a band at 650–850 nm, while larger SB particles have a broader band at 900–950 nm, as expected for bulky gold materials (Fig. 3f).

### Enhanced dark-field images for the visualization of particles in cells

Next, we characterized the particles in A549 and HepG2 cells. Cells were fixed after 24 h of treatment, and dark-field imaging was utilized to image the particles in cells with high resolution. Using this system, the noise-to-signal ratio is up to ten times lower than that of other optical instruments, providing enhanced nanoscale images of nanomaterials^36,37^. In this system, the oblique angle of source illumination is adjusted such that it precisely focuses on the sample and bypasses the objective. This provides high contrast (very intense scatter from the sample and very dark background) to detect particles in cells.

**Fig. 4:**
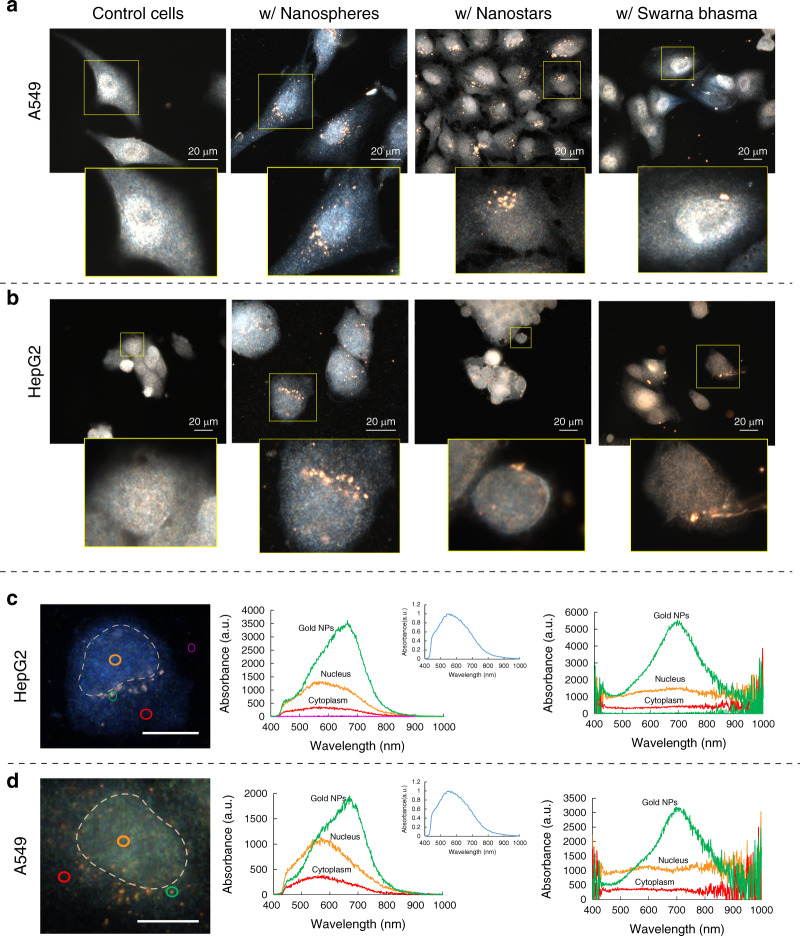
Dark-field images of cells incubated with gold nanoparticles and their intracellular LSPR. **a** Dark-field images of A549 cancer cells treated with three different types of particles. **b** Dark-field images of HepG2 cancer cells treated with three different types of particles. Few SBs entered into cells, while nanospheres were found more often in cells. **c** Spectral responses from different regions of HepG2 cells (nucleus and cytoplasm), gold nanospheres in HepG2 cells, and corrected responses after normalization with respect to the halogen lamp spectrum (right panel). **d** Spectral responses from different regions of A549 cells (nucleus and cytoplasm), gold nanospheres in A549 cells, and corrected responses after normalization with respect to the halogen lamp spectrum (right panel). The scale bar is 20 µm

Figure 4a, b shows DK images of cells with the different gold particles compared to nontreated cells. Due to the dark background, the gold particles are bright and easy to visualize. A549 and HepG2 cells had more nanospheres than nanostars or SBs. As shown in the DK images, nanospheres were localized mostly in the perinuclear regions. A549 cells appeared to contain more nanospheres than HepG2 cells. Nanostars were more randomly distributed in the cytosol in A549 and HepG2 cells, while cells had much fewer SBs. These larger particles may be more challenging for cells to take up^30^. Therefore, in contrast to nanospheres and nanostars, SB particles were not found in all cells, and they were relatively randomly distributed, similar to nanostars.

### Particle detection with hyperspectral methods

As discussed earlier, HSIs can be used to detect particles such as gold NPs in cells. In Fig. 4c, d, typical HSI images of A549 and HepG2 cells are shown. Figure 4c, d show the absorbance spectra from different regions of the cell, including the cytoplasm (red) and nucleus (orange). The absorbance intensity of the nucleus was higher than that of the cytoplasm. The nucleus is more electron-dense than the cytosol and likely absorbs more light. In Fig. 4c, d, the green profiles show the absorbance of the gold particle/aggregate in cells. Due to their specific LSPR properties, gold NPs display unique spectral information depending on their size and morphology, as well as their surrounding microenvironment. The band position of NPs in cells and how it changes can be used to detect them at the subcellular level. A large peak is seen between 600–700 nm corresponding to the gold nanoparticles. The cytoplasm and nucleus have peaks in the range of 500–600 nm, which comes from the light source (shown in Fig. 4c, d–inset). We normalized the HSI profiles to obtain more precise spectral responses. The normalized data are plotted in Fig. 4c, d (right panels). After correction, the spectra from the nucleus and cytoplasm are flat, and the gold particle/aggregate peak is at 700 nm. The corrected spectral information allows the detection of particles in cells and characterizes their properties in cells.

### Particle mapping in hyperspectral images

Different particles in cells might not be detected visually with HSI images. Scanning through the spectral data of an HSI image can be utilized to distinguish pixels that match our desired spectra. The spectral angle mapper algorithm was used to scan HSI images spectrally. Before performing SAM analysis, a spectral reference was defined for gold particles to classify the different pixels in the HSI images. With SAM, the spectral similarity is measured between two spectra. With this algorithm, each spectrum was assumed to be a vector (with a direction and length), and the angle between the vector at each pixel and the F-SPL vector (gold particles) was calculated and compared to the threshold. With the SAM algorithm, we could detect pixels whose bands are similar to gold NPs and diagnose particles that are not visible in HSI images. This technique can be used to determine the distribution/localization of particles within cells.

**Fig. 5:**
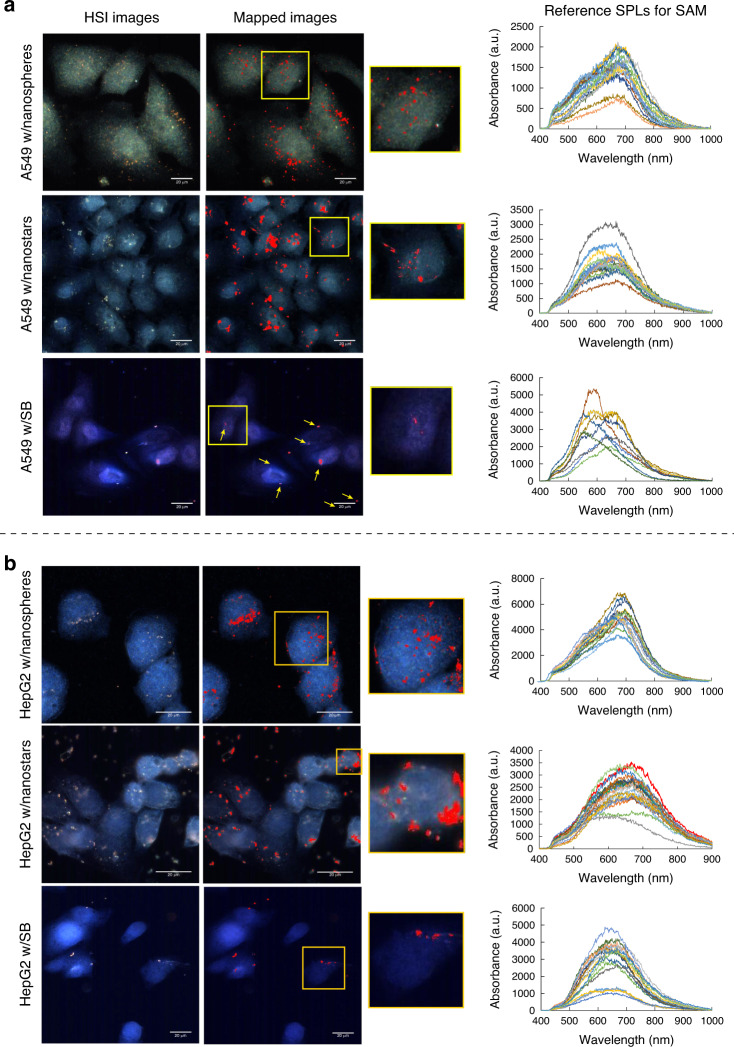
Spectrally mapping of gold nanoparticles in cells using the spectral angle mapper algorithm. **a** Spectrally mapped gold particles in A549 cells with the SAM algorithm against the SPL (right panel) to localize particles in different regions of cells, **b** Spectrally mapped gold particles in HepG2 cells with the SAM algorithm against the SPL (right panel) to localize particles in different regions of HepG2 cells. Scale bar is 20 µm

Due to the lack of resolution of HSI images compared to DK images, spectral measurements were used to detect the particles. Figure 5a shows the mapping of three different types of particles in A549 cells with respect to SPL shown on the right panel. With the SAM algorithm, the particles are labeled in red after mapping to show their distribution in cells. Nanospheres were enriched in perinuclear regions, although they could be seen in various regions of the cell, including the nucleus. Zooming into a single cell, the mapped image shows nanospheres in the nucleus, although they were not visible in the original HSI image. Nanostars were more aggregated and localized more randomly than nanospheres, with only a few in the nucleus. SB particles are larger, and the mapped image does not provide further information on their distribution in cells. As shown in the right panel, their spectra varied more, likely due to their irregular morphology. Similar mapping was performed on HepG2 cells, as shown in Fig. 5b. The distribution patterns of the three different types of gold particles in HepG2 cells were similar to those in A549 cells. HepG2 cells had more nanospheres and nanostars than SB particles. However, HepG2 cells appeared to have fewer nanospheres and nanostars than A549 cells. There was no significant change in SB distributions between the two cell lines, and only small aggregates were in cells, with larger particles on the membrane surface. We observed a few individual/aggregates of nanospheres in the HepG2 nucleus, and their level of internalization was less than that of A549 cells.

### NP spectral responses for intracellular diagnosis

Plasmon particles can also be used for intracellular sensing and the imaging of biomarkers in cells. Different strategies have been employed to take advantage of the optical properties of plasmon particles to detect/visualize subcellular components and biomarkers in cells. For example, Kumar et al.^38^ utilized functionalized gold NPs to bind actin biomarkers in cells to provide strong molecular-specific optical signals. As mentioned earlier, plasmon particles can also enhance Raman signals to provide SERS probes for intracellular detection and imaging. All these techniques rely on optical enhancement because of the LSPR properties of NPs. In addition to optical enhancements, plasmon NPs can be used as sensing tools to detect biomarkers based on their LSPR changes. Plasmonic or LSPR shifts have been widely used to develop biosensors; however, to the best of our knowledge, this technique has not been used for intracellular diagnosis. The LSPR of gold NPs is influenced by size, morphology, and the local microenvironment. The LSPR scattering maximum wavelength is sensitive to the microenvironment and its optical properties, such as the dielectric constant or refractive index. Therefore, any change in the local environment of gold NPs can cause a shift in the LSPR band (plasmonic shift). This change can be quantified through the following equation^39,40^:

1 $$\Delta \lambda _{{\mathrm{max}}} = m\Delta n\left[ {1 - {\mathrm{exp}}\left( {\frac{{ - 2d}}{{l_d}}} \right)} \right]$$

where Δ*λ*_max_ is the maximum expected plasmonic shift, m is the bulk refractive index of the NPs, Δ*n* is the refractive index change induced by the absorbance, *d* is the thickness of the dielectric layer, and *l*_*d*_ is the characteristic electromagnetic-field-decay length. This equation shows that the plasmonic shift is directly proportional to changes in the dielectric constant of the local environment. By increasing the dielectric layer thickness, the last term is increased, causing a higher shift in the LSPR band.

**Fig. 6:**
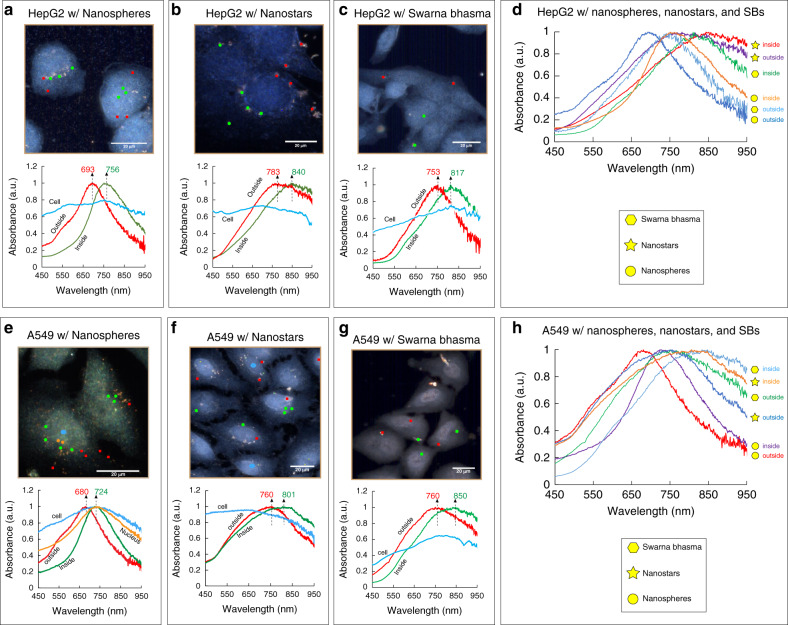
Spectral responses of gold particles in different regions of both cell lines (outside or periphery and inside). **a** HepG2 cells with sphere-shaped particles showing a plasmonic shift from 693 nm to 756 nm for outside and inside particles. **b** HepG2 cells with nanostars, showing a plasmonic shift from 783 nm to 840 nm for outside and inside particles. **c** HepG2 cells with SB particles and the plasmonic shift from 753 nm to 817 nm. **d** Spectra of three different morphologies of particles in different regions of HepG2. **e** A549 cells with sphere-shaped particles showing a plasmonic shift from 680 nm to 724 nm for outside and inside particles. **f** A549 cells with nanostars, showing a plasmonic shift from 760 nm to 801 nm. **g** A549 cells with SB particles and the plasmonic shift from 760 nm to 850 nm. **h** Spectra of three different morphologies of particles in different regions of A549 cells. Scale bar is 20 µm

Shifts in the LSPR band of gold NPs in cells enable us to detect and sense small changes in the environment adjacent to particles and verify their presence in cells. Different regions in cells exhibit different microenvironments, which affect the optical responses of biomolecules or particles in their vicinity^41^. These effects are reflected in the LSPR responses. In Fig. 6a, b, and c, the LSPR bands of three different types of particles in HepG2 cells are illustrated. Gold particles were marked with different colors in different regions of cells (edge/outside of cells in red, cytosol in green), and their corresponding spectra were compared in the bottom panel. As shown in Fig. 6a, gold nanospheres outside cells or in the periphery of cells exhibited peaks at ~693 nm. Nanospheres inside cells had a shifted spectrum of 756 nm. This relatively large shift is likely due to differences in the surrounding environment. This shift might also be related to other parameters, including their interactions with intracellular components, such as proteins and/or lipids, or changes in abiotic parameters, such as intracellular pH. For this large shift, however, changes in the microenvironment seem to be more significant than interactions with biochemical components^42^. Particles interact with different subcellular compartments, so the local environment surrounding particles is dramatically changed, altering the optical properties of their local regions. The effects of these changes can be reflected in the spectral responses. As shown in Fig. 6a, the LSPR for particles situated outside HepG2 cells is sharper, while for inside particles, it becomes broader, likely due to differences in the microenvironment. Spectra are also influenced by the size and aggregation of particles. The size of nanospheres is more consistent than the other two types, and their plasmonic shifts can be solely correlated to their intracellular interactions and environments. However, for SBs and nanostars, the effects of size and degree of aggregation should be taken into account in interpreting their plasmonic shifts. For nanostars, the bands shifted from 760–790 nm to 830–860 nm for particles localized at the periphery and inside HepG2 cells, respectively (Fig. 6b). SB particles are larger, and only a few smaller particles were inside the cells. Their bands shifted similar to those of nanospheres but were broader (Fig. 6c). Due to the irregular morphology and varying size of SBs, their LSPR changes may also be associated with their size and morphology.

A similar comparison was made for A549 cells with the three different types of particles to characterize their interactions with subcellular components. Since the nuclear region was clearer in these images, particles in the nucleus were also measured (orange). For gold nanospheres, the peak shifted to the right from 680 nm to 724 nm for particles outside/in the periphery of cells compared to particles in the cytosol (Fig. 6e). The LSPR band of particles in the nucleus was similar to that of particles in the cytosol but broader (full width at half maximum). The LSPR of smaller particles is sensitive than that of larger particles^43^ to the surrounding region/surface, so they can reflect changes in their environment better. Therefore, the smaller size of nanospheres allows them to better sense their surrounding changes, and they appeared to be more sensitive to changes in their local environment. Another noticeable change in spectral responses was the peak broadness. The broadness was increased by 10–15% for nanospheres situated in the cells compared to nanospheres localized at the periphery of cells (Fig. 6e). The broadness change (%) was determined by measuring the increase or decrease in the LSPR peak width (at half maximum) of NPs inside cells compared to the NPs outside or in the periphery of cells. In contrast to nanostars and SBs, a few nanospheres were also found in the nucleus of A549, and as shown in Fig. 6e, their plasmonic bands were shifted while they became broader compared to the NPs in the cytosol. In addition to the different interactions of NPs with subcellular compartments, the differences in dielectric constants could be another reason for the plasmonic shifts between particles situated in the periphery and particles in the cytoplasm or the nucleus. Some studies show that the cytoplasm of cells has dielectric constants ten times higher than that of the cell membrane^41,44^, and in another study^45^, the dielectric constant of the nucleus was found to be four times higher than that of the cytoplasm. Nanostars tend to aggregate, and their LSPR peaks were broader than those of nanospheres. Their peaks shifted from 760 nm to 801 nm for particles/aggregates outside cells compared to those inside cells (Fig. 6f). SBs are larger, and only a few were found inside cells where they randomly localized in the cytosol. The mean band for particles inside cells shifted from 760 nm (outside) to 850 nm (inside), and the peaks were broad, likely reflecting their nonuniform morphologies (Fig. 6g).

The plasmonic shifts for different cells and particles are reported in Table 1. The plasmonic shift takes place for all three types of particles between 41 and 90 nm depending on the type of cells, the morphology of particles, and their subcellular location. The data in Table 1 indicate a more consistent behavior of nanospheres in terms of peak broadness and the plasmonic shift, while two other particles exhibited different peak broadnesses in two different cell lines, likely due to the nonidentical shapes of SBs and the different aggregation levels of nanostars. The spectral profiles of nanostars revealed that they aggregated at the periphery and inside the cells. Therefore, nanostar plasmonic shifts from outside to inside cells can reflect both particle-particle interactions and particle-subcellular interactions. The results also indicated that the plasmonic shifts are stronger for SB particles whose size is larger compared to other particles. It seems that higher shifts in SBs originate mostly from the induced changes between particles rather than interactions with subcellular systems. Among these three particles, nanospheres are the best candidates to reveal information on the local environment due to their effective interactions with their surrounding medium. Any shifts in their bands can be correlated to the different subcellular regions of cells with better accuracy than two other particles. In addition, nanospheres offer higher quality factors (Q-factor) than the two other types of particles (Table 1). The quality factor was calculated as the ratio between the LSPR peak (nm) and the width at half maximum (nm)^46^. The quality factor shows the energy losses for the coherent motion of the electron and depends on the size, morphology, and surrounding medium of the NPs. For surrounding medium sensing, resonances with higher quality factors are desirable (narrow widths)^47–49^. Moreover, our results showed that SB particles and nanostars have limitations in reaching different subcellular regions of cells. However, due to their smaller sizes, nanospheres were found in different subcellular regions in cells, enabling their use for subcellular diagnosis through intracellular plasmonics.

**Table 1:** Effects of the surrounding medium and morphology of NPs on intracellular plasmonics

Cell line		Nanospheres	Nanostars	Swarna Bhasma
		Spherical	Branched	Nonuniform
A549 cells	Plasmonic shift (Δ*λ*)^a^	~44 ± 9 nm	~41 ± 7 nm	~90 ± 14 nm
	Broadness change^b^	+13.6%	−8.9%	+19.1%
	Q-factor (inside cells)^c^	4.55	2.68	4.45
HepG2 Cells	Plasmonic shift	~57 ± 8 nm	~48 ± 10 nm	~54 ± 10 nm
	Broadness change	+9.1%	+19.6%	+4.3%
	Q-factor	4.72	2.46	3.82

^a^$$\Delta \lambda = \lambda _{{\mathrm{inside}}} - \lambda _{{\mathrm{outside}}}$$

^b^$\Delta w = 100 \times \frac{{w_{{\mathrm{inside}}\,{\mathrm{cytoplasm}}} - w_{{\mathrm{periphery}}\,{\mathrm{of}}\,{\mathrm{cells}}}}}{{w_{{\mathrm{periphery}}\,{\mathrm{of}}\,{\mathrm{cells}}}}}$ ($w$: full width at half maximum)

^c^$Q = \frac{\lambda }{w}$ (*λ*: peak frequency, $w$: full width at half maximum)

### Particle distribution in cells

**Fig. 7:**
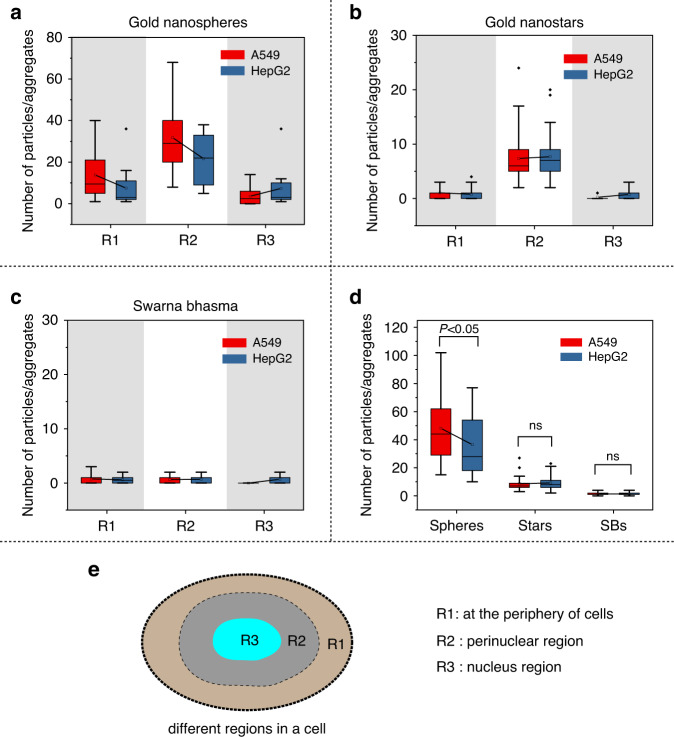
Gold nanoparticles depending on their nanomorphology and size, are distributed differently in different regions of cells. **a** Nanosphere particle distribution in different regions of cells for both cell lines, **b** gold nanostar distribution in different regions of cells for both cell lines, **c** SB particle distributions in cells for both cell lines, **d** the total number of particles in two cell lines, **e** different regions in cells (R1: far from the nucleus and at the cell periphery, R2: perinuclear region, and R3: nucleus)

After entering cells, particles were localized in three different regions: at the periphery or distant from the nucleus, in the perinuclear region, and inside the nucleus. Discrete nanospheres could be identified in different parts of cells, while nanostars aggregated either inside cells or at their periphery. SB particles are much larger, the size of their aggregates might reach one micron, and their internalization level was not high. To estimate the number of particles in cells, the occupied area of particles in cells was measured, and by considering the average size of particles, they were counted in cells. At least 150 cells were considered for each type of particle, and their distributions were quantified in three different (Fig. 7e) regions and plotted in Fig. 7.

The average number of nanospheres per cell was higher for both cell lines than the two other types of particles, and A549 cells had almost two times more nanospheres. Note that A549 cells are larger in comparison to HepG2 cells, and they could accommodate more particles. There was no significant difference between the particle numbers of the two other particles in the two cell lines. In contrast to nanostars and nanospheres, the uptake of SB particles in cells was very low, and their distributions in different regions were random. It is evident that particles (nanospheres and nanostars) were mostly localized in cells and at the periphery of the nucleus (Fig. 7a, b). This pattern can be seen in both cell lines. SB particles were few (with smaller size) in cells and did not follow a specific distribution pattern in cells.

## Conclusions and outlook

In NP-based cancer therapy, precise subcellular detection/distribution of NPs is crucial to designing effective nanomedicines. Although the detection of NPs in biological environments has been accomplished using electron microscopy, newer, cheaper, more advanced, and faster methodologies provide opportunities. The highly sensitive optical properties of gold NPs have provided a novel and noninvasive platform to develop LSPR-based methods to diagnose NPs at subcellular levels. In this work, this idea was verified using three different gold particles with different morphologies. We showed how hyperspectral imaging and spectral measurements at the nanoscale enable us to approximate their localization in different subcellular regions and understand their interactions with subcellular compartments. Intracellular diagnosis with hyperspectral imaging allows not only confirmation of the presence of particles in cells but also nanoscale spectral measurements to provide information on their surrounding medium. Inside the cell, there are various microenvironments, and gold particles localized in these regions exhibit different plasmonic shifts depending on the optical properties of their surrounding regions, as well as their physicochemical properties. Our results revealed that particles could sense their intracellular environments. We examined particle responses at different subcellular regions, at the periphery of cells, at the perinuclear and, inside the nucleus. Our measurements showed that particles, depending on their interactions with their local environments, have different intracellular plasmonic responses. During these interactions, the surrounding medium of particles is changed, affecting the LSPR properties of NPs. Stability and consistency in both morphology and size of particles are two important factors for subcellular detection. Due to the inconsistency and irregular shape of SBs and the strong tendency of nanostars to aggregate, the plasmonic shift in their responses cannot be purely correlated to modifications in their surrounding mediums and how they interact with subcellular compartments. The effects of particle-particle interactions and the morphology of particles could also be reflected by plasmonic shifts. Among the three particles, nanospheres were distributed more uniformly within cells and appeared to reflect the effects of their surrounding environments better, as they showed a more consistent behavior in cells. Furthermore, the distribution of particles showed that nanospheres are capable of navigating to different subcellular regions in cells, while the two other particles had limited access to different subcellular regions. Designing proper NPs with specific physicochemical properties will play a critical role in subcellular diagnosis with hyperspectral imaging. For future works, the unique spectral signature of gold NPs and plasmonic shifts of NPs can be used to create a comprehensive library for different subcellular regions/organelles of cells for more accurate subcellular sensing. This is feasible when the HSI-based subcellular diagnostic technique is simultaneously used with high-resolution microscopic techniques to visualize subcellular regions/organelles with more details. This library can be used to detect NPs at subcellular levels, accurately predict their localizations in cells, and study how they interact with their subcellular compartments.
